# Surveillance and epidemiology of syphilis, gonorrhoea and chlamydia in the non-European Union countries of the World Health Organization European Region, 2015 to 2020

**DOI:** 10.2807/1560-7917.ES.2022.27.8.2100197

**Published:** 2022-02-24

**Authors:** Jelena Barbaric, Giorgi Kuchukhidze, Nicole Seguy, Elena Vovc, Maria Josefina Theresa Babovic, Teodora Elvira Wi, Daniel Low-Beer, Ivana Bozicevic

**Affiliations:** 1 World Health Organization Collaborating Centre for HIV Strategic Information, University of Zagreb School of Medicine, Zagreb, Croatia; 2 World Health Organization Regional Office for Europe, Copenhagen, Denmark; 3 World Health Organization Headquarters, Geneva, Switzerland

**Keywords:** sexually transmitted infections, surveillance, syphilis, gonorrhoea, chlamydia, antimicrobial resistance, Europe

## Abstract

**Background:**

Epidemics of sexually transmitted infections (STI) are a major public health challenge in the World Health Organization (WHO) European Region.

**Aim:**

We aimed to provide an overview of case reporting and other surveillance data for syphilis, gonorrhoea and chlamydia for the non-European Union (EU)/European Economic Area (EEA) countries of the Centre and East part of the WHO European Region as per classification used by the WHO Regional Office for Europe (WHO/Europe) and the European Centre for Disease Prevention and Control.

**Methods:**

Data were provided by the surveillance agencies of the Member States for the period 2015 to 2019 through the WHO/Europe Communicable Diseases Annual Reporting Form. We analysed reported cases, explored data reported to the WHO Gonococcal Antimicrobial Surveillance Programme (GASP) and performed a review of publications on antimicrobial resistance (AMR) in gonorrhoea in the period 2015 to 2020 using systematic methodology.

**Results:**

From 2015 to 2019, in most of the countries with three or more data points, there was a pattern of decrease in reported syphilis, gonorrhoea and chlamydia cases, which is in contrast to the EU/EEA. The number of reported cases per 100,000 population was 0.4–26.5 for syphilis, 0–18.5 for gonorrhoea and 0–43.3 for chlamydia. Four countries reported recent data on AMR in gonorrhoea to GASP, and we identified further publications from Georgia, Russia and Ukraine.

**Conclusion:**

We found wide heterogeneity in reported rates of STI. There is a strong need to improve availability and quality of STI surveillance data in the non-EU/EEA countries.

## Introduction

Control of sexually transmitted infections (STI) is a major public health challenge in the World Health Organization (WHO) European Region [1,2]. Without adequate treatment, STI may have serious long-term health repercussions, such as reproductive health complications and increased risk of acquiring HIV or cancers. In 2016, 10.7% of women aged 15–49 years in the WHO European Region were estimated to have a urogenital infection with herpes simplex virus type 2 (HSV-2), 3.2% with *Chlamydia trachomatis*, 1.6% with *Trichomonas vaginalis*, 0.3% with *Neisseria gonorrhoeae* and 0.1% with *Treponema pallidum*, whereas prevalence estimates among 15–45 year-old men were somewhat lower [1,2].

Monitoring of progress towards the targets of the WHO Global Health Sector Strategy on STI requires implementation of robust strategic information systems [3,4]. Furthermore, the current COVID-19 pandemic may undermine the progress towards the Global Strategy targets as it seems to disproportionally affect key and vulnerable populations at risk of HIV and other STI, primarily because of limited access to healthcare services[5].

STI surveillance is essential for the development of STI programmes, advocacy and management of cases [4]. Core components of STI surveillance include case reporting, STI prevalence surveys in populations at higher (e.g. female sex workers (FSW) or men who have sex with men (MSM)) and at lower risk (e.g. pregnant women and young people), assessment of STI syndrome aetiologies and assessment of antimicrobial resistance (AMR) patterns [4]. Epidemiological data on STI are available for 30 European Union (EU)/European Economic Area (EEA) countries that participate in the European STI Surveillance Network and report through the European Surveillance System (TESSy). However, information is scarce from the non-EU/EEA countries of the WHO European Region. A systematic review by Rowley et al. did not identify any published studies on chlamydia or on gonorrhoea prevalence in the period from 2009 to 2016 among the general population in the non-EU/EEA countries of the WHO European Region. Through personal communication, the authors identified one unpublished dataset from 2012 among antenatal clinic attendees aged 14–44 years in Georgia (n = 300), with a chlamydia prevalence of 5.0% and a gonorrhoea prevalence of 0.3% [1]. Monitoring progress in STI control in the non-EU/EEA countries has wider implications. The analysis of European Gonococcal Antimicrobial Surveillance Programme (Euro-GASP) data collected from 2010 to 2014 showed that those born in non-EU/EEA countries of the WHO European Region had the highest rates of resistance to ciprofloxacin (71.7%), azithromycin (11.2%) and cefixime (9.1%), and the second highest rate of decreased susceptibility to ceftriaxone (8.7%), compared with isolates of patients from the EU/EEA countries and all other WHO Regions [6].

The aims of this paper were to provide an overview of the following for the non-EU/EEA countries of the WHO European Region: (i) characteristics of case reporting systems for syphilis, gonorrhoea and chlamydia, (ii) number of reported cases of syphilis, gonorrhoea and chlamydia and rate per 100,000 population for 2015 to 2019, reported to the WHO/Europe Communicable Diseases Annual Reporting Form (CDARF), (iii) laboratory tests used to diagnose syphilis, gonorrhoea and chlamydia, (iv) results of a review on AMR in gonorrhoea and (v) gaps in STI surveillance and laboratory diagnostics and priority actions for improvements in STI surveillance in the Region.

## Methods

We included in this assessment 18 non-EU/EEA countries of the Centre and East region as per the classification from WHO and the European Centre for Disease Prevention and Control (ECDC): Albania, Armenia, Azerbaijan, Belarus, Bosnia and Herzegovina, Georgia, Kazakhstan, North Macedonia, Kyrgyzstan, Republic of Moldova, Montenegro, Serbia, Tajikistan, Turkey, Turkmenistan, the Russian Federation, Ukraine and Uzbekistan [7]. We also included data from Kosovo* which were available from the information sources listed below.

### Information sources for existing components of surveillance systems for sexually transmitted infections

In October 2020, the WHO Regional Office for Europe (WHO/Europe) sent a questionnaire to the focal points for STI surveillance in the countries to assess the existing components of their STI surveillance systems (the questionnaire is available as Supplement 1). We sought information about the types of case reporting (universal or sentinel) for syphilis, gonorrhoea, chlamydia, lymphogranuloma venereum (LGV), genital herpes and HPV infection, modalities of reporting (mandatory or voluntary) and availability of information on modes of transmission (MoT) and site of infection for cases of syphilis, gonorrhoea and chlamydia. We asked respondents to estimate the coverage of STI reporting (the proportion of diagnosed STI cases actually reported to central level), as previously assessed for the EU countries [8].

We assessed the availability of STI prevalence studies covering the general population and of screening programmes for chlamydia in young people (age group: 15–24 years) and opportunistic testing for chlamydia. In addition, the same questionnaire from October 2020 sought information about laboratory tests used to diagnose syphilis, gonorrhoea or chlamydia. For this purpose, we used and adapted selected questions from the ECDC survey done in 2013 for the EU countries [9].

### Sources of data on sexually transmitted infections

We used the most recent period for each of the data sources from 2015 onwards. Data on reported cases of syphilis, gonorrhoea and chlamydia for the period 2015 to 2019 were extracted from the WHO/Europe Communicable Diseases Annual Reporting Form (CDARF). The CDARF collects annual case-based data on diseases that can cause epidemics, including STI, in the WHO European Region, through section 2 of the Joint Reporting Form (JRF) that was developed jointly by WHO and the United Nations International Children's Emergency Fund (UNICEF) in an effort to strengthen collaboration and minimise the reporting burden of all Member States. Data on congenital syphilis and prevalence of active syphilis in FSW, MSM and male sex workers (MSW) were extracted for the period 2017 to 2019 from the Global AIDS Monitoring (GAM) database of the Joint United Nations Programme on HIV/AIDS (UNAIDS), namely GAM 2018, GAM 2019 and GAM 2020 [10-12].

To obtain information about surveillance of AMR in gonorrhoea, we explored data from 2015 to 2018 of the WHO GASP, available through the WHO World Health Data Platform [13]. In addition, we performed a literature review using a systematic review methodology to identify reports on AMR in gonorrhoea in the countries included in the assessment. We developed, registered and followed a standardised protocol (PROSPERO ID: CRD42021229042) for this purpose where detailed methodological approach is described [14]. We searched PubMed, Embase and Web of Science databases for articles published between 1 January 2015 and 31 December 2020. We also screened references of included reports for eligible publications. We did not apply any language restrictions. Our search strategies are available in Supplement 2 Table S1. We included reports which contained data on decreased susceptibility or resistance (DS/R) to ceftriaxone, azithromycin, cefixime, extended-spectrum cephalosporins or ciprofloxacin, and other antibiotics from one or more isolates of *Neisseria gonorrhoeae* collected between 2015 and 2020. We extracted information on study design, number of isolates, study population (age, sex, risk group), anatomical site of specimen collection, pathogen identification, antimicrobial susceptibility testing methods and antimicrobial DS/R data. We also assessed the quality of published studies using adapted criteria published by Tadesse et al. [15]. Two researchers (JB and IB) independently screened reports for inclusion and extracted data in line with the protocol. Disagreements were resolved by a third researcher (GK).

### Data analysis

We analysed reported cases of syphilis, gonorrhoea and chlamydia by sex and calculated rates of reported cases per 100,000 population in the period 2015 to 2019. Furthermore, we calculated proportions of young persons (persons aged 15–24 years) among all confirmed cases of syphilis, gonorrhoea and chlamydia older than 15 years with information on age reported in WHO EURO CDARF. Population data for the calculation of rates were extracted for each year from the United Nations World Population Prospects [16]. 

We performed a narrative synthesis of the data on AMR in gonorrhoea identified in the WHO GASP and through the literature review using systematic methodology [14].

### Ethical statement

Ethical approval was not necessary since we used secondary data sources and already published data.

## Results

### Characteristics of case reporting systems and availability of prevalence data for sexually transmitted infections in the general population

Seven countries provided responses about availability and characteristics of STI case reporting and STI prevalence assessment surveys in general and key populations collected via the WHO/Europe questionnaire. Six countries completed the questionnaire on laboratory diagnostics.

Universal reporting from public and private healthcare providers was the source of data on syphilis, gonorrhoea and chlamydia in Belarus, Georgia, Montenegro, North Macedonia, Serbia and Ukraine. There was universal reporting of LGV in Belarus and Serbia, whereas in Ukraine LGV was reported from sentinel sites (Table 1). In Kazakhstan, syphilis and gonorrhoea were subject to universal reporting from public providers, while chlamydia was reported from both public and private providers. The estimated coverage of case reporting was highest for syphilis, followed by gonorrhoea (Table 1). Of note is the limited inclusion of LGV in case reporting systems (Table 1).

**Table 1:** Estimated percentage of syphilis, gonorrhoea, chlamydia and lymphogranuloma venereum cases that are reported, seven WHO European Region countries, 2020

	Syphilis (%)	Gonorrhea (%)	Chlamydia (%)	Lymphogranuloma venereum (%)
Belarus^a^	76–100	76–100	51–75	76–100
Georgia^b^	76–100	76–100	76–100	Not reported
Kazakhstan^b^	76–100	51–75	51–75	Not reported
Montenegro^b^	< 25	< 25	< 25	Not reported
North Macedonia^b^	< 25	< 25	26–50	Not reported
Serbia^b^	76–100	26–50	26–50	< 25
Ukraine^c^	90	50	50	Sentinel

STI: sexually transmitted infections; WHO: World Health Organization.

^a^ Syphilis, gonorrhoea and lymphogranuloma venereum are subject to mandatory reporting while chlamydia is reported on a voluntary basis.

^b^ All mentioned STI are reported on a mandatory basis.

^c^ Reporting is done on a voluntary basis.

Information about transmission category and the site of infection for cases of syphilis, gonorrhoea and chlamydia was not available in Georgia, Montenegro, North Macedonia, Serbia and Ukraine. Data disaggregated by age and sex were collected in Belarus, Georgia, Montenegro, North Macedonia, Serbia and Ukraine. In Belarus, information about MoT was collected for syphilis, gonorrhoea and LGV, and for the site of infection for syphilis and gonorrhoea. In Kazakhstan, MoT information was reportedly available only for syphilis, while information about the site of infection was not collected.

In Georgia, Kazakhstan, Montenegro, North Macedonia and Serbia, there were no data on the prevalence of chlamydia in young people (defined as 15–24 year-olds), while they were reportedly available from facility-based surveys in Ukraine and Belarus. Screening programmes for chlamydia in young people reportedly existed only in Belarus at the local level. In Montenegro opportunistic testing for chlamydia existed only in fertility clinics but data were not available to the WHO/Europe focal point. In Belarus and Ukraine, opportunistic testing for chlamydia for young people was available at family planning and reproductive health clinics, clinics for STI and fertility clinics. Data from these testing services were reported in Belarus and Ukraine at national level.

### Case reporting data for syphilis, gonorrhoea and chlamydia

Data from the CDARF on reported syphilis cases were available for 15 countries in the period 2015 to 2019 (Table 2). The highest syphilis case rate in the last year we included (2018 or 2019 depending on data availability) was observed in Georgia (26.5 cases per 100,000 population), followed by Kazakhstan (18.8/100,000) and the Russian Federation (16.6/100,000). In these countries and in Belarus, where also more than two data points were available, the rates appeared to be declining in the period from 2015 to 2019.

**Table 2:** Reported cases of syphilis and case notification rate per 100,000 population by country, 15 WHO European Region countries, 2015–2019

	2015		2016		2017		2018		2019	
	Number	Rate	Number	Rate	Number	Rate	Number	Rate	Number	Rate
Albania	NA		NA		56	1.9	68	2.4	NA	
Armenia	NA		NA		55	1.9	NA		110	3.7
Azerbaijan	560	5.7	908	9.2	967	9.8	981	9.9	824	8.2
Belarus	686	7.3	565	6.0	NA		NA		405	4.3
Bosnia and Herzegovina	NA		15	0.5	33	1.1	NA		NA	
Georgia	1,316	32.8	1,349	33.7	NA		1,243	31.1	1,059	26.5
Kazakhstan	NA		4,703	26.4	4,516	26.1	3,774	20.6	3,484	18.8
Kyrgyzstan	676	11.3	371	6.1	NA		NA		NA	
Montenegro	0	0	1	0.1	3	0.5	NA		NA	
North Macedonia	1	0.05	4	0.2	4	0.2	8	0.4	NA	
Russian Federation	34,465	23.8	31,050	21.4	NA		24,144	16.6	NA	
Serbia	146	1.6	159	1.8	162	1.8	206	2.3	NA	
Tajikistan	NA		NA		NA		360	4.0	NA	
Ukraine	NA		NA		NA		NA		2,486	5.7
Uzbekistan	NA		2,326	7.4	2,267	7.1	2,697	8.3	2,874	8.7

NA: data not available; WHO: World Health Organization.

Age-disaggregated data on syphilis cases were available for 11 countries. Young people contributed with the greatest proportion of cases in Serbia (21.7–32.7%), Kyrgyzstan (23.1%) and Kazakhstan (21.1-21.9%) in 2015 to 2019. The smallest proportions were reported from Armenia, Belarus and Georgia, ranging from 7.2% to 9.1% in the most recent year with available data.

Data on congenital syphilis available in the GAM database for the period 2017 to 2019 for 11 countries indicated that the highest reported case rate was in Georgia, ranging from 14.7 to 20.8 cases per 100,000 live births during this time period. This is substantially below the global target for elimination of mother-to-child transmission of syphilis, which is fewer than 50 cases per 100,000 live births.

The same 15 countries that reported on syphilis cases reported also on gonorrhoea in the period 2015 to 2019 (Table 3). The highest gonorrhoea case notification rate in 2018 or 2019, the last year with available data, was observed in Georgia (18.5/100,000), and rates were above 10 per 100,000 population in Kazakhstan and Uzbekistan. There was a pattern of decrease in the most recently reported rates in the countries that had available data for at least 3 years, with the exception of Azerbaijan.

**Table 3:** Reported cases of gonorrhoea and case notification rate per 100,000 population by country, 15 WHO European Region countries, 2015–2019

	2015		2016		2017		2018		2019	
	Number	Rate	Number	Rate	Number	Rate	Number	Rate	Number	Rate
Albania	NA		NA		4	0.1	8	0.3	NA	
Armenia	NA		NA		308^a^	10.5	NA		291	9.8
Azerbaijan	537	5.6	439	4.5	415	4.2	530	5.3	306	3.0
Belarus	2,048	21.7	1731	18.3	NA		NA		767	8.1
Bosnia and Herzegovina	2^a^	0.1	4^a^	0.1	1^a^	0	NA		NA	
Georgia	712	17.7	923	23.0	NA		765	19.1	738	18.5
Kazakhstan	NA		3,506	19.7	2,912	16.1	2,552	13.9	1,950	10.5
Kyrgyzstan	703	11.8	215	3.5	NA		NA		NA	
Montenegro	10	1.6	4	0.6	4	0.6	NA		NA	
North Macedonia	5	0.2	5	0.2	4	0.2	0	NA	NA	
Russian Federation	27,042	18.7	21,072	14.5	NA		12,733	8.7	NA	
Serbia	87	1.0	103	1.2	81	0.9	71	0.8	NA	
Tajikistan	NA		NA		NA		243	2.7	NA	
Ukraine	NA		NA		NA		NA		3,263	7.4
Uzbekistan	NA		4,155^a^	13.2	3,923	12.3	3,575^a^	11.0	3,540^a^	10.7

NA: data not available; WHO: World Health Organization.

^a^ Not reported whether laboratory-confirmed.

Age-disaggregated data on gonorrhoea cases were available for 14 countries. The greatest proportion of cases in young people in countries with more than 10 reported cases annually was seen in Belarus (43.7–45.6%), Armenia (41.6–34.9%), Uzbekistan (40.2% in 2017) and Kazakhstan (32.6–37.0%). The smallest percentage in 2019 was reported from Ukraine (22.6%). We observed a pattern of a decline in the proportions of cases in young people among the reported gonorrhoea cases in Georgia (34.2–23.0%), whereas an increase was noted in Azerbaijan (19.6–33.7%) and a stable pattern in Kazakhstan (around 35%) and in Belarus (around 45%). The greatest fluctuation in the proportional contribution of young people to the total number of cases was seen in the countries with the smallest number of cases reported (Bosnia and Herzegovina and Montenegro).

As shown in Table 4, the highest rate of reported chlamydia in the last included year (2018 or 2019) was observed in Georgia (52.0/100,000), Belarus (43.3/100,000) and Ukraine (31.6/100,000). In Belarus, Georgia and Kazakhstan, the rate of reported cases decreased throughout the studied period.

**Table 4:** Reported cases of chlamydia and case notification rate per 100,000 population by country, 15 WHO European Region countries, 2015–2019

	2015		2016		2017		2018		2019	
	Number	Rate	Number	Rate	Number	Rate	Number	Rate	Number	Rate
Albania	NA		NA		0	0	0	0	NA	
Armenia	NA		NA		838^a^	28.5	NA		779	26.3
Azerbaijan	2,115	22.0	3,153	32.4	1,548	15.7	2,244	22.6	1,910	19.0
Belarus	7,144	75.7	5,659	59.9	NA		NA		4,095	43.3
Bosnia and Herzegovina	72^a^	2.1	67^a^	2.0	197^a^	5.9	NA		NA	
Georgia	2,293	57.0	2,507	62.4	NA		2,083	52.0	1,559	39.0
Kazakhstan	NA		3,484	19.5	3,284	18.2	3,101	16.9	2,506	13.5
Kyrgyzstan	1,736	29.1	NA		NA		NA		NA	
Montenegro	5	0.8	17	2.7	15	2.4	NA		NA	
North Macedonia	264	12.7	124	6.0	137	6.6	95	4.6	NA	
Serbia	972	10.9	897	10.1	713	8.1	879	10.0	NA	
Tajikistan	NA		NA		NA		47	0.5	NA	
Ukraine	NA		NA		NA		NA		13,907	31.6

NA: data not available; WHO: World Health Organization.

^a^ Not reported whether laboratory confirmed.

Age-disaggregated data on chlamydia cases were available for 11 countries. Young people contributed more than 30% of chlamydia cases in Armenia, Belarus and Tajikistan. In Georgia, we observed a declining pattern in the proportion of young people among the total number of cases (41.4–21.0%). In Montenegro and North Macedonia, there was substantial fluctuation in the proportion of reported cases among young people across the years. 

Of note are wide differences in the reported rates of STI across countries. The most recent data indicated that the number of reported cases per 100,000 population ranged from 0.4 to 26.5 for syphilis, from 0 to 18.5 for gonorrhoea and from 0 to 43.3 for chlamydia.

Most of the countries reported more cases of syphilis and gonorrhoea in men than in women (Figure). The male-to-female ratio was higher for gonorrhoea than for syphilis, except in Armenia and Serbia. In Kazakhstan, Kyrgyzstan and Uzbekistan there were minor or no differences in the proportion of male and female syphilis cases. In contrast, the proportion of reported cases of chlamydia in women outnumbered those in men in the majority of countries with the exception of Azerbaijan.

**Figure:**
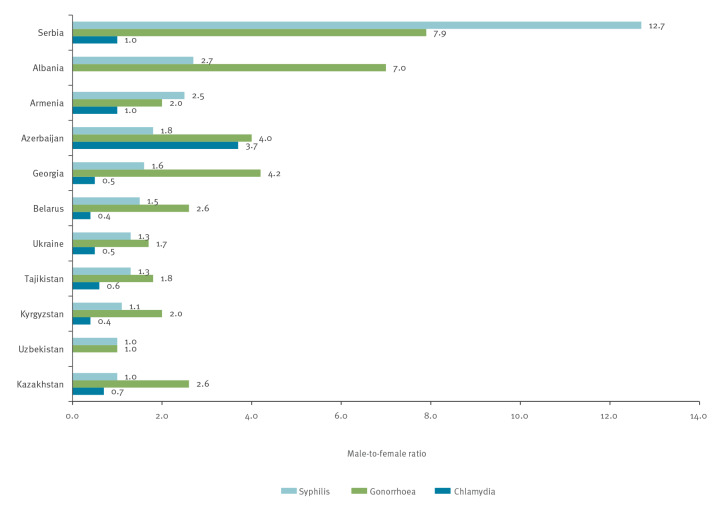
Male-to-female ratio in reported cases of syphilis, gonorrhoea and chlamydia, 11 WHO European Region countries, most recent year with available data in the 2015–2019 period

WHO: World Health Organization.

### Syphilis prevalence in key populations from the Global AIDS Monitoring database

Prevalence data on active syphilis in FSW were submitted to GAM in 2017 to 2019 by nine countries, data for MSW by two and for MSM by 10. Data on MSW were collected in very small samples of fewer than 50 MSW. The syphilis prevalence among FSW ranged from 0% in Kosovo* to 20.9% in Azerbaijan (the median reported syphilis seroprevalence in FSW was 5.7%). In MSM, syphilis prevalence ranged from 0.5% in Ukraine and Armenia to 13.3% in Moldova, and the median prevalence was 3.8%.

### Laboratory methods to diagnose syphilis, gonorrhoea and chlamydia

In the six countries that supplied responses to the questionnaire section on laboratory methods, conventional laboratory techniques (*Treponema pallidum* haemagglutination assay (TPHA), *T. pallidum* particle agglutination assay (TPPA), enzyme immunoassays (EIA)) were used as screening tests (Table 5). In all six countries, rapid plasma reagin (RPR), venereal disease research laboratory tests (VDRL) and IgM treponemal antibodies tests were also performed. Nucleic acid amplification tests (NAAT)) for *N. gonorrhoeae* is available, mainly for genital samples, and *N. gonorrhoeae* cultures were also performed. In all six countries, the diagnostic techniques needed to meet the WHO and EU laboratory criteria for the case confirmation of syphilis, congenital syphilis and gonorrhoea for surveillance purposes were reportedly performed [4,17-19].

**Table 5:** Laboratory methods to diagnose syphilis, gonorrhoea and chlamydia, six WHO European Region countries, 2015–2019

	Belarus	Georgia	Kazakhstan	Montenegro	Serbia	Ukraine
Laboratory methods used to confirm a case of syphilis						
Detection of *T. pallidum* antibodies by screening test (TPHA, TPPA or EIA)	+	+	+	+	+	+
RPR/VDRL	+	+	+	+	+	+
Detection of IgM antibodies to *T. pallidum*	+	+	+	+	+	+
Demonstration of *T. pallidum* in lesion exudates or tissues	+	+	+	−	−	+
- Dark-field microscopic examination	+	+	+	−	−	+
- Direct fluorescent antibody *T. pallidum* test	−	−	+	−	−	−
- Molecular detection	−	−	−	−	−	+
Other	+^a^	−	−	−	+^b^	−
Laboratory methods used to confirm a case of congenital syphilis						
Demonstration of *T. pallidum* in the umbilical cord, the placenta, a nasal discharge or skin lesion material	−	+	−	−	−	+
- Dark-field microscopic examination	−	+	−	−	−	+
- Direct fluorescent antibody *T. pallidum* test	−	−	−	−	−	−
Detection of *T. pallidum*-specific IgM	+	+	+	+	+	+
Reactive non-treponemal test (VDRL, RPR) in the baby's/child’s serum	+	+	+	+	+	+
Other	+^a^	-	-	-	+^c^	+^d^
Laboratory methods used to confirm a case of gonorrhoea						
Isolation and confirmation of *N. gonorrhoeae* from a clinical specimen	+	-	+	+	+	+
Detection of *N. gonorrhoeae* nucleic acid in a clinical specimen	+	+	+	+	+	+
- Genital samples	+	+	+	+	+	+
- Rectal samples	+	+	-	-	-	+
- Pharyngeal samples	+	-	-	-	-	+
Demonstration of *N. gonorrhoeae* by a non-amplified nucleic acid probe test in a clinical specimen	−	−	−	−	−	−
Microscopic detection of intracellular Gram-negative diplococci in an urethral male specimen	+	+	+	+	+	+
Identification of culture	+	+	+	+	+	+
- Gram	+	+	+	+	+	+
- Oxidase	+	+	-	+	+	+
- Biochemical tests	-	+	-	-	-	+
- Molecular tests	+	-	+	-	+	+
Other	−	−	−	−	−	−
Laboratory methods used to confirm a case of chlamydia						
Isolation of *C. trachomatis* from a specimen of the anogenital tract or from the conjunctiva	+	+	-	-	-	-
Demonstration of *C. trachomatis* by DFA test in a clinical specimen	+	+	+	-	+	+
Detection of *C. trachomatis* nucleic acid in a clinical specimen	+	+	+	+	+	+
- Genital samples	+	+	+	+	+	+
- Rectal samples	+	+	-	-	-	+
- Pharyngeal samples	+	-	-	-	-	+
Other methods	+^e^	+^f^	-	-	-	-

+: method available; −: method not available; *C. trachomatis*: *Chlamydia trachomatis*; DFA: direct fluorescent antibody; EIA: enzyme immunoassays; *N. gonorrhoeae*: *Neisseria gonorrhoeae*; RPR: rapid plasma reagin; *T. pallidum*: *Treponema pallidum*; TPHA: *T. pallidum* haemagglutination assay; TPPA: *T. pallidum* particle agglutination assay; VDRL: venereal disease research laboratory test; WHO: World Health Organization.

^a^ Passive hemagglutination reaction, indirect immunofluorescence, immunoblotting performed when the results of other treponemal tests are questionable or contradictory.

^b^ Line blot.

^c^ TPHA.

^d^ In most laboratories, a non-treponemal test used is microprecipitation reaction (modification of VDRL).

^e^ Culture (for women only), immunofluorescence reaction.

^f^ EIA.

Similar to gonorrhoea, NAAT to diagnose *C. trachomatis* was available in all six countries, but testing for extragenital samples was limited. Direct fluorescent antibody (DFA) tests were commonly applied. Isolation from a specimen of the anogenital tract or from the conjunctiva is one of the methods accepted as laboratory criteria for the WHO and the EU case confirmation of chlamydia, but it seems to be available in only two countries [4,17-19]. Information on the proportion of reported cases of syphilis, gonorrhoea and chlamydia that are confirmed with specific diagnostic methods and their combinations is not available.

### Surveillance of gonococcal antimicrobial susceptibility

In the period from 2015 to 2018, only four non-EU/EEA countries reported AMR and susceptibility testing data of at least one drug to GASP, namely Belarus, Kyrgyzstan, the Russian Federation and Ukraine (Supplement 2 Table S2 shows the reported numbers of tested isolates and the test results). Analysis of AMR data for more than 100 gonococcal isolates in a year in GASP was available only from the Russian Federation in 2015 and 2016. Through literature review using systematic methodology, we identified seven relevant records corresponding to five studies of gonococcal isolates in Georgia [20], Ukraine [21,22] and the Russian Federation [23-26] (a flow chart of searches and details about these studies are listed in Supplement 2 Figure S1 and Table S2). We assessed an additional five full text articles and excluded them, with reasons provided in Supplement 2 Table S3 [27-31].

Our analysis of the GASP data from 2015 to 2018 did not find any reported isolates with resistance to azithromycin, nor DS/R to ceftriaxone. The reported proportion of isolates with DS/R to both cefixime and extended-spectrum cephalosporins in Belarus was 22.2% in 2017 and 15.8% in 2018. The other three countries did not report any isolates with DS/R to cefixime or to extended-spectrum cephalosporins. In Belarus, the proportion of isolates with resistance to ciprofloxacin was 30.6% in 2017 and 36.8% in 2018. In Ukraine, 10% of isolates were resistant to ciprofloxacin in 2018 and 21.4% in 2017, while the Russian Federation did not report any isolates with resistance to ciprofloxacin to GASP in the observed period.

We identified a single published case report from Georgia in 2018 which presented resistance to cefixime and ciprofloxacin identified in a urethral specimen of a 23-year-old heterosexual man [20]. Data from Ukraine, reported to GASP, were also included in two publications by Boiko et al. [21,22]. We identified several articles from the Russian Federation that covered different time periods [23-26]. In a study by Kubanov et al. that included 124 gonorrhoea strains collected from nine regions in 2015, resistance (R) in *N. gonorrhoeae* to ceftriaxone was 0.8%, intermediate susceptibility (IS) to azithromycin 3.2%, R to azithromycin 1.6%, IS to ciprofloxacin 0.8%, and to ciprofloxacin 40.3% [23]. Another report covering 522 samples from 16 regions during the period 2015 to 2017 found three isolates (0.6%) with DS to ceftriaxone, but it was unclear in which year exactly the isolates with DS had been collected [26]. A report on 268 specimens from 2016 did not find resistance to ceftriaxone, but only 91.4% of specimens were susceptible to azithromycin and 31% were resistant to ciprofloxacin [25]. All those studies found during literature review published AMR data that were based on testing only urethral specimens from male and cervical/urethral specimens from female patients. We were not able to find AMR testing results disaggregated by age, sex and sexual orientation.

The reviewed studies are detailed in Supplement 2 Table S2. They reported laboratory methods more consistently and in greater detail than epidemiological data on the study population relevant for surveillance purposes, as explained in the quality assessment of the studies in Supplement 2 Table S4.

## Discussion

Our overview of surveillance systems and associated laboratory methods for syphilis, gonorrhoea and chlamydia in the non-EU/EEA European countries of the WHO European Region highlights the need for strengthening of surveillance and laboratory diagnostics. Availability of STI data varied substantially among the countries included in this assessment and they were not standardised and thus comparable only to a limited extent. Overall, with this limitation in mind, we noted a substantial difference in trends of syphilis, gonorrhoea and chlamydia when this region is compared with the EU/EEA. However, information on MoT of reported cases, necessary for appropriate interpretation of epidemiological data on STI, was mainly not collected, which makes it difficult to draw conclusions about STI epidemics in these countries.

The highest rate of reported syphilis infection in 2018 and 2019 was observed in several countries of the East (Georgia, Kazakhstan, Russian Federation, Belarus) but there was a decrease in reported rates. For comparison, the crude notification rate of syphilis in the EU/EEA countries was 7.0 per 100,000 population in 2018, while the highest rates were recorded in Malta (17.9/100,000), Luxembourg (17.1/100,000) and the United Kingdom (UK) (12.6/100,000) [32]. Overall, there seemed to be a higher number of reported syphilis cases in the East part of the WHO European Region (as per WHO/ECDC classification: West, Centre, East) and a lower number in the Centre, mirroring similar patterns in HIV case reporting [7]. In addition, the male-to-female ratio for syphilis cases in 2019 in the reporting countries of the East included in this assessment ranged from 1.0 to 1.8, which is similar to the average male-to-female ratio for HIV in 2019 (1.6), as published in the HIV/AIDS surveillance report for Europe across the EU/EEA countries [7]. In the Centre, the male-to-female ratio for syphilis was available only for Albania and Serbia (2.7 and 12.7, respectively) compared with the average of 5.5 for HIV across the countries of the Centre [7]. However, it should be acknowledged that injecting drug use was a dominant mode of HIV transmission in 24% of HIV cases newly reported in the East in 2019, while this was observed in 4% of cases in the Centre. Syphilis prevalence data among key populations in GAM were available for some countries and varied considerably. These differences are probably in part due to the use of different sampling and testing methods which our findings revealed. On the other hand, STI epidemics are local, and our findings also point to the importance of regular data collection in key populations using consistent methods in individual countries.

In all the countries included in our assessment, the rates of gonorrhoea in the period 2015 to 2019 were lower than the crude notification rate in the EU/EEA countries in 2018 (26.4/100,000). The gonorrhoea case rate has been increasing since 2009 in those EU/EEA countries reporting consistently, which is in contrast to a decline observed in the majority of the non-EU/EEA countries [33].

The overall male-to-female ratio of syphilis and gonorrhoea cases in 2018 in the EU/EEA countries was 8.5 and 3.2, respectively, which is on average higher compared with the countries included in our study (median value of 1.5 for syphilis and 2.6 for gonorrhoea) [33]. These findings might indicate greater presence of syphilis in the general population in the non-EU/EEA countries with the available data, or lower likelihood of MSM to be tested for syphilis and reported.

The chlamydia case reporting rate in the countries included in this analysis was overall considerably lower than in the 23 EU/EEA countries in 2018 (146 per 100,000 in the countries with comprehensive surveillance systems) [33]. These differences might be due to a range of issues such as lower coverage of reporting in the non-EU/EEA countries, provision of STI services to vulnerable populations by private providers who are less likely to report, limited screening coverage and the use of suboptimal diagnostics. A recent study in Ukraine identified suboptimal sensitivity of conventional diagnostic tests for chlamydia, gonorrhoea and trichomoniasis, and a need for widespread implementation of validated and quality-assured molecular diagnostic tests [34].

The number of countries included in this assessment which participate in the WHO GASP is small as was the availability of published data on AMR in gonorrhoea. It is encouraging that four of these countries reported that there was no resistance to ceftriaxone in 2015 and 2016, although it is noteworthy that a high level of cefixime resistance was found in Belarus and of cefixime-resistant isolates were detected in Kyrgyzstan [35] and Georgia [20]. Resistance to ceftriaxone of less than 1% is reported in published literature from Russia [25,26]. We were able to identify both GASP data and relevant studies only for Ukraine and the Russian Federation. There was a clear and matching overlap of data for Ukraine from different sources, whereas we noted some discrepancies in the data available for the Russian Federation. Furthermore, some of the published reports were of insufficient quality (e.g. no information on date of sample collection; see Supplement 2 Table S4 for details), which makes the interpretation even more challenging. According to the most recent data of EURO GASP, the overall cefixime and azithromycin resistance in the EU/EEA has been stable since 2014 at around 2% and 7–8%, respectively, while in 2017, no isolate resistant to ceftriaxone was detected [36]. Overall, owing to a lack of more recent AMR data, the extent of the spread of resistant gonococcal strains remains unknown. The majority of published AMR data for gonorrhoea in the non-EU/EEA countries contained limited epidemiological characteristics such as sex, age, sexual orientation and site of infection, resulting in an inability to understand the patterns of resistance. Studies included in the review reported AMR data in isolates only from urethral and cervical specimens.

Overall, the surveillance of gonococcal antimicrobial susceptibility should be improved in non-EU/EEA countries by increasing the number of countries participating in the WHO GASP, ensuring that countries provide an adequate number of isolates, linking epidemiologic data with laboratory data and strengthening epidemiological and laboratory capacities for the surveillance of gonococcal antimicrobial susceptibility [37].

All of the countries reported the use of laboratory methods meeting the WHO and EU case definitions for syphilis, gonorrhoea and chlamydia. Importantly, it remains unclear what proportion of cases are diagnosed using specific methods. We also found that testing of rectal and pharyngeal samples was uncommon. The NAAT have generally better sensitivity than culture [38], in particular for pharyngeal and rectal samples, but also higher costs and potentially suboptimal specificity, so the samples tested may need a supplementary NAAT, which, together with the fact that rectal and pharyngeal samples are reportedly not performed in many countries, may explain lower male-to-female ratios in the non-EU/EEA compared with the EU/EEA countries where NAAT are used more commonly [33,38]. Furthermore, NAAT may be more commonly performed in private laboratories and clinics, which are less likely to report diagnosed cases.

Interpretation of STI data is influenced by their completeness and representativeness and by the laboratory methods used in STI diagnostics. Our results should therefore be interpreted with caution, taking the context of the setting into account (see Supplementary Table S5 for a summary of contextual factors). Only a minority of countries provided data through CDARF, GAM and GASP. In addition, seven of 18 eligible countries provided responses to the WHO/Europe questionnaires, so those results cannot be generalised further. Among these, only two countries showed a reporting rate > 75% for syphilis and gonorrhoea and only one country for chlamydia. This implies some uncertainty regarding the reliability of the data. Differences in case definitions among individual countries may be another limitation common for studies comparing data from different countries [8]. However, the WHO criteria for case confirmation expected when reporting through CDARF make the data comparable to a certain extent. The questionnaire we used collected information on laboratory methods needed to meet those criteria and, considering that only seven countries provided information, asking for exact case definitions in individual countries could have added more detail but would probably have led to even fewer countries responding.

The comparisons among the non-EU/EEA and EU/EEA have to be interpreted with caution owing to the heterogeneity in reporting and healthcare systems in the countries, as well as the fact that large chlamydia screening programmes were conducted in some countries such as the UK. Underlying factors such as access to testing, in particular for key populations, possible underdiagnosis and under-reporting influence the interpretation of the data and the differences among individual countries, and need further in-depth exploration in future studies.

## Conclusion

Among the key findings of this analysis is a still rather limited availability of STI data, including AMR data in gonorrhoea, in tools that the WHO uses for STI data collection and reporting such as CDJRF, GAM and GASP. Moving forward, STI surveillance systems and laboratory diagnostics needs to be further strengthened so as to better estimate the burden of STI, measure progress towards the STI control targets set by the WHO and guide the implementation of national STI control programmes. Specific to the European Region is the importance of availability of STI trend data in key populations, in particular in MSM in the context of pre-exposure prophylaxis for prevention of HIV transmission and decreased condom use.

## *Note

This designation is without prejudice to positions on status, and is in line with UNSCR 1244/99 and the ICJ Opinion on the Kosovo declaration of independence.

## Supplementary Data

420000 Supplement1

## Supplementary Data

204000 Supplement2
