# Supplementary material for: Surveillance and epidemiology of syphilis, gonorrhoea and chlamydia in the non-European Union countries of the World Health Organization European Region, 2015 to 2020
Source: Euro Surveill. 2022 Feb 24;27(8):2100197. doi: 10.2807/1560-7917.ES.2022.27.8.2100197 (PMC8874864; doi:10.2807/1560-7917.ES.2022.27.8.2100197)
Supplement: Supplement1 [file 21-00197_BOZICEVIC_Supplement1_Questionnaire.pdf]

SUPPLEMENTARY MATERIAL 1: QUESTIONNAIRE

This supplementary material is hosted by *Eurosurveillance* as supporting information alongside the article "*Surveillance and epidemiology of syphilis, gonorrhoea and chlamydia in the non-European Union countries of the World Health Organization European Region*", on behalf of the authors, who remain responsible for the accuracy and appropriateness of the content. The same standards for ethics, copyright, attributions and permissions as for the article apply. Supplements are not edited by *Eurosurveillance* and the journal is not responsible for the maintenance of any links or email addresses provided therein.

World Health Organization

Assessment of STI surveillance systems in non-EU countries of WHO European Region

PROJECT ID:

Date form completed:

Day	Month	Year
		2 0 2 0

This questionnaire will take approximately 30 minutes to complete. Thank you for taking the time.

This questionnaire seeks information about operational characteristics of certain components of STI surveillance systems and the types of laboratory tests that are most commonly used to diagnose STIs.

Name of Country: ______________________________

Name of Person or Persons Completing this Form: ______________________________

Job titles/description:

1= National STI programme director or manager
2= National HIV programme director or manager
3= National sexual and reproductive health officer or director
4= National programme officer for maternal and child health
5= National disease surveillance coordinator
6= National STI surveillance officer or manager
7= WHO country programme officer
8= Other UN agency HIV, STI or sexual and reproductive health officer (UNICEF, UNAIDS, UNFPA)
9= Other

If ***Other***, please specify: ______________________________

E-mail address: ______________________________

STI case reporting

1. Is reporting of the following STIs universal or sentinel?

Universal reporting implies that all health care providers that diagnose STIs are expected to report.

Sentinel reporting means that reporting is done only be selected health care facilities.

	Universal, includes public and private providers	Universal, includes only public providers	Sentinel
a) Syphilis	☐	☐	☐
b) Gonorrhoea	☐	☐	☐
c) Chlamydia	☐	☐	☐
d) Lymphogranuloma venereum (LGV)	☐	☐	☐
e) Trichomoniasis	☐	☐	☐
f) Genital herpes	☐	☐	☐
g) Human papillomavirus (HPV)	☐	☐	☐

2. If reporting of the following STIs is universal, approximately what percentage of cases are actually reported?

Please check the box	<25%	26-50%	51-75%	76-100%	Not universal
a) Etiologies: Syphilis	☐	☐	☐	☐	☐
Syphilis in pregnant women	☐	☐	☐	☐	☐
b) Etiologies: Gonorrhoea	☐	☐	☐	☐	☐
c) Etiologies: Chlamydia	☐	☐	☐	☐	☐
d) Etiologies: LGV	☐	☐	☐	☐	☐
e) Etiologies: Trichomoniasis	☐	☐	☐	☐	☐
f) Etiologies: Genital herpes	☐	☐	☐	☐	☐
g) Etiologies: HPV	☐	☐	☐	☐	☐

3. Is reporting of the following STIs compulsory or voluntary?

	Compulsory	Voluntary
a) Syphilis	☐	☐
b) Gonorrhoea	☐	☐

c) Chlamydia	☐	☐
d) LGV	☐	☐
e) Trichomoniasis	☐	☐
f) Genital herpes	☐	☐
g) HPV	☐	☐

4. For which STI case reports is information available on transmission category?

(men who have sex with men, heterosexual, mother-to-child, unknown, etc).

	Yes	No
a) Syphilis	☐	☐
b) Gonorrhoea	☐	☐
c) Chlamydia	☐	☐
d) LGV	☐	☐
e) Trichomoniasis	☐	☐
f) Genital herpes	☐	☐
g) HPV	☐	☐

5. Are STI case reporting data evaluated annually for:

	Yes	No
a) completeness of information on reporting forms	☐	☐
b) timeliness of reporting	☐	☐
c) accuracy of reporting	☐	☐

6. Is information on site of infection (genital, rectal, oral) in individual case reporting form available?

	Yes	No
a) Syphilis	☐	☐
b) Gonorrhoea	☐	☐
c) Chlamydia	☐	☐

7. Is information on sex and age in individual case reporting form available?

	Yes	No
a) Syphilis	☐	☐
b) Gonorrhoea	☐	☐
c) Chlamydia	☐	☐

STI prevalence assessment

8. Were STI prevalence assessment surveys conducted among general population of men and women since 2015?
1= Yes 2= No 3= Information unknown ☐

If yes, please indicate in which populations and which STIs were tested for

	syphilis	gonorrhoea	chlamydia	trichomonas	genital herpes
a) general population household-based surveys	☐	☐	☐	☐	☐
b) pregnant women	☐	☐	☐	☐	☐
b) women attending family planning clinics	☐	☐	☐	☐	☐
c) military recruits	☐	☐	☐	☐	☐
d) work-based health screening programmes	☐	☐	☐	☐	☐
e) other - please indicate _________________	☐	☐	☐	☐	☐

9. Testing for which STIs was included in the last round of HIV bio-behavioural surveys in key and vulnerable populations?

1=Yes 2= No 3= Information unknown

	MSM	Sex workers	PWID	Migrants	TGW
a) Syphilis					
b) Gonorrhoea					
c) Chlamydia					
d) Genital herpes					
e) Hepatitis B					
f) Hepatitis C					
g) other					

10. In which year were the above mentioned studies done?

MSM				
FSW				
PWID				
Migrants				
TGW				

11. Are there data available on prevalence of Chlamydia trachomatis infection in young people?

Yes ☐ No ☐

If yes, please indicate the data source and the most recent year for which data are available

a) General population-based surveys in young people Year ☐
b) School or university-based surveys Year ☐
c) Health care facility-based surveys
(includes clinical sites such as school health clinics, reproductive health clinics, etc)
d) Data collected from screening or opportunistic testing in health care facilities, schools, etc
e) Other - please indicate ________________

12. Is there a screening programme available for genital Chlamydia trachomatis infection in young people (age groups 16-24)

By a screening programme, we mean a continuous organised service where chlamydia tests are regularly offered to a defined population at a high enough coverage to benefit the population.
Screening programmes can be register-based, i.e. people are invited from a maintained register (e.g. population register or healthcare register) to take a test, or opportunistic, i.e. professionals offer a test to eligible people in a predetermined setting

a) No ☐

b) Yes, there is a national-level screening programme for C trachomatis infection ☐

c) Yes, there are regional or local level screening programmes for C trachomatis infection ☐

13. At which faciities or sites is opportunistic testing for chlamydia available?

*Opportunistic chlamydia testing refers to chlamydia tests which are offered to people in predefined risk groups who have **no symptoms.***

Groups to whom opportunistic testing can be provided are young people, pregnant women, female sex workers, men who have sex with men, etc.

	Yes	No
a) General practices	☐	☐
b) Clinics for sexually transmitted infections	☐	☐
c) Family planning and reproductve health clinics	☐	☐
d) Fertility clinics	☐	☐
e) Schools	☐	☐
f) Universities	☐	☐
g) Via Internet (tests can be ordered on-line)	☐	☐

h) Other - please indicate ____

14. If there is a screening programme or opportunistic testing for chlamydia available, are data on the number of chlamydia cases diagnosed and number of diagnostic tests done reported to the national level public health/ surveillance agency?

a) Yes ☐

b) No ☐

Organisation of the STI diagnostic service

15. How many laboratories perform STI diagnostics in your country? Please give numbers ifknown, and please feel free to comment.

Number of public laboratories: ☐ Information unknown ☐
Comments:

Number of private laboratories: ☐ Information unknown ☐
Comments:

16. Please indicate the type of expert/specialist function of laboratories available in your country for gonorrhoea,

chlamydia and syphilis. Please feel free to comment.

National: *Receives isolates or specimens from the whole country/acts as a national reference centre*
Regional: *Receives isolates or specimens from an area within the country/acts as a regional reference centre*
Expert: *is not designated as a reference centre but receives isolates or specimens from other laboratories in the whole country or area within the country*
Routine: *Laboratory performs routine diagnostics for STIs and does not have an expert/specialist function*

Please check all that apply.

	National	Regional	Expert	Routine
Gonorrhoea:	☐	☐	☐	☐
Chlamydia:	☐	☐	☐	☐
Syphilis:	☐	☐	☐	☐

If applicable, please specify areas they cover (e.g. county, oblast etc.) for Regional/Expert:

Comments: ____

____

17. What proportion of laboratories which perform STI diagnostics in your country are accredited or registered? Please specify the accrediting body (bodies) and feel free to comment.

<25%	26-50%	51-75%	76-100%	Information unknown
☐	☐	☐	☐	☐

Accrediting body (bodies) and any comments: ______________________________

____

Laboratory diagnostics*

Please check the box(es) . If yes, please specify the proportion of reported cases in 2019 for which the method applies. If the information is unknown, please insert "NA".

18. Please indicate which laboratory methods are used to confirm a case of gonorrhoea in your country

	Yes	No	% in 2019
18.1 Isolation and confirmation of N. gonorrhoeae from a clinical specimen	☐	☐	☐
18.2 Identification of culture is performed by:			
a) Gram	☐	☐	
b) Oxidase	☐	☐	

c) Biochemical tests

d) Immunological tests

e) Molecular tests

18.3 Detection of Neisseria gonorrhoeae nucleic acid in a clinical specimen

a) Genital samples tested

b) Rectal samples tested

c) Phryngealsamples tested

18.4 Demonstration of N. gonorrhoeae by a non-amplified nucleic acid probe test in a clinical specimen

18.5 Microscopic detection of intracellular gram negative diplococci in an urethral male specimen

18.6 Other laboratory methods used to confirm a case of gonorrhoea
Please specify:

Comments: ____

____

19. Please indicate which laboratory methods are used to confirm a case of chlamydial infection in your country.

(all serovars including L)

	Yes	No	% in 2019
19.1 Isolation of C. trachomatis from a specimen of the ano-genital tract or from the conjunctiva	☐	☐	☐
19.2 Demonstration of C. trachomatis by DFA test in a clinical specimen	☐	☐	☐
19.3 Detection of C. trachomatis nucleic acid in a clinical specimen	☐	☐	☐
a) Genital samples tested	☐	☐	
b) Rectal samples tested	☐	☐	
c) Phryngealsamples tested	☐	☐	
19.4 Other laboratory methods used to confirm a case of chlamydial infection	☐	☐	☐

Please specify: ______________________________

Comments: ______________________________

Yes No

20. Is dual molecular testing for gonorrhoea and chlamydia available in your country? ☐ ☐

Comments: ______________________________

21. Please indicate which laboratory methods are used to confirm a case of syphilis in your country

	Yes	No	% in 2019
21.1 a) Detection of T. pallidum antibodies by screening test (TPHA, TPPA or EIA)<br>If yes, please specify which test:	☐	☐	☐
AND additionally:			
b) RPR (VDRL). If yes please specify:	☐	☐	☐
c) Detection of IgM antibodies to Treponema pallidum	☐	☐	☐
21.2 Demonstration of T. pallidum in lesion exudates or tissues by dark-field microscopic examination			
21.3 Demonstration of T. pallidum in lesion exudates or tissues by DFA test	☐	☐	☐
21.4 Molecular detection of T. pallidum in lesion exudates or tissues			
21.5 Other laboratory methods used to confirm a case of syphilis<br>Please specify:	☐	☐	☐

Comments: ______________________________

22. Please indicate which laboratory methods are used to confirm a case of congenital syphilis in your country

	Yes	No	% in 2019
22.1 Demonstration of T. pallidum by dark field microscopy in the umbilical cord, the placenta, a nasal discharge or skin lesion material	☐	☐	
22.2 Demonstration of T. pallidum by DFA-TP in the umbilical cord, the placenta, a nasal discharge, or skin lesion material	☐	☐	
22.3 Detection of T. pallidum - specific IgM	☐	☐	
22.4 Reactive non treponemal test (VDRL, RPR) in the baby/child's serum	☐	☐	
22.5 Other laboratory methods used to confirm a case of congenital syphilis<br>Please specify:	☐	☐	

Comments: ____

____

* *Questions adapted from European Centre for Disease Prevention and Control. STI laboratory diagnostics in Europe. Stockholm: ECDC; 2013 Stockholm: ECDC; 2013*
