# Supplementary material for: Surveillance and epidemiology of syphilis, gonorrhoea and chlamydia in the non-European Union countries of the World Health Organization European Region, 2015 to 2020
Source: Euro Surveill. 2022 Feb 24;27(8):2100197. doi: 10.2807/1560-7917.ES.2022.27.8.2100197 (PMC8874864; doi:10.2807/1560-7917.ES.2022.27.8.2100197)
Supplement: Supplement2 [file 21-00197_BOZICEVIC_Supplement2.pdf]

SUPPLEMENTARY MATERIAL 2

This supplementary material is hosted by *Eurosurveillance* as supporting information alongside the article "*Surveillance and epidemiology of syphilis, gonorrhoea and chlamydia in the non-European Union countries of the World Health Organization European Region"*, on behalf of the authors, who remain responsible for the accuracy and appropriateness of the content. The same standards for ethics, copyright, attributions and permissions as for the article apply. Supplements are not edited by *Eurosurveillance* and the journal is not responsible for the maintenance of any links or email addresses provided therein.

Supplementary Table 1. Search strategies for searches up to *8 January 2021*

PubMed
Gonorrhea[Mesh] OR Gonorrhea[Text Word] OR Neisseria gonorrhoeae[Mesh] OR gonococcus[Text Word] AND Drug Resistance, Bacterial[Mesh] OR antibacterial drug resistance[Text Word] AND Albania<br>Gonorrhea[Mesh] OR Gonorrhea[Text Word] OR Neisseria gonorrhoeae[Mesh] OR gonococcus[Text Word] AND Drug Resistance, Bacterial[Mesh] OR antibacterial drug resistance[Text Word] AND Armenia<br>Gonorrhea[Mesh] OR Gonorrhea[Text Word] OR Neisseria gonorrhoeae[Mesh] OR gonococcus[Text Word] AND Drug Resistance, Bacterial[Mesh] OR antibacterial drug resistance[Text Word] AND Azerbaijan<br>Gonorrhea[Mesh] OR Gonorrhea[Text Word] OR Neisseria gonorrhoeae[Mesh] OR gonococcus[Text Word] AND Drug Resistance, Bacterial[Mesh] OR antibacterial drug resistance[Text Word] AND Belarus<br>Gonorrhea[Mesh] OR Gonorrhea[Text Word] OR Neisseria gonorrhoeae[Mesh] OR gonococcus[Text Word] AND Drug Resistance, Bacterial[Mesh] OR antibacterial drug resistance[Text Word] AND Bosnia and Herzegovina<br>Gonorrhea[Mesh] OR Gonorrhea[Text Word] OR Neisseria gonorrhoeae[Mesh] OR gonococcus[Text Word] AND Drug Resistance, Bacterial[Mesh] OR antibacterial drug resistance[Text Word] AND Georgia<br>Gonorrhea[Mesh] OR Gonorrhea[Text Word] OR Neisseria gonorrhoeae[Mesh] OR gonococcus[Text Word] AND Drug Resistance, Bacterial[Mesh] OR antibacterial drug resistance[Text Word] AND Kazakhstan<br>Gonorrhea[Mesh] OR Gonorrhea[Text Word] OR Neisseria gonorrhoeae[Mesh] OR gonococcus[Text Word] AND Drug Resistance, Bacterial[Mesh] OR antibacterial drug resistance[Text Word] AND Kosovo<br>Gonorrhea[Mesh] OR Gonorrhea[Text Word] OR Neisseria gonorrhoeae[Mesh] OR gonococcus[Text Word] AND Drug Resistance, Bacterial[Mesh] OR antibacterial drug resistance[Text Word] AND Macedonia<br>Gonorrhea[Mesh] OR Gonorrhea[Text Word] OR Neisseria gonorrhoeae[Mesh] OR gonococcus[Text Word] AND Drug Resistance, Bacterial[Mesh] OR antibacterial drug resistance[Text Word] AND Kyrgyzstan<br>Gonorrhea[Mesh] OR Gonorrhea[Text Word] OR Neisseria gonorrhoeae[Mesh] OR gonococcus[Text Word] AND Drug Resistance, Bacterial[Mesh] OR antibacterial drug resistance[Text Word] AND Moldova<br>Gonorrhea[Mesh] OR Gonorrhea[Text Word] OR Neisseria gonorrhoeae[Mesh] OR gonococcus[Text Word] AND Drug Resistance, Bacterial[Mesh] OR antibacterial drug resistance[Text Word] AND Montenegro<br>Gonorrhea[Mesh] OR Gonorrhea[Text Word] OR Neisseria gonorrhoeae[Mesh] OR gonococcus[Text Word] AND Drug Resistance, Bacterial[Mesh] OR antibacterial drug resistance[Text Word] AND Serbia<br>Gonorrhea[Mesh] OR Gonorrhea[Text Word] OR Neisseria gonorrhoeae[Mesh] OR gonococcus[Text Word] AND Drug Resistance, Bacterial[Mesh] OR antibacterial drug resistance[Text Word] AND Tajikistan<br>Gonorrhea[Mesh] OR Gonorrhea[Text Word] OR Neisseria gonorrhoeae[Mesh] OR gonococcus[Text Word] AND Drug Resistance, Bacterial[Mesh] OR antibacterial drug resistance[Text Word] AND Turkey<br>Gonorrhea[Mesh] OR Gonorrhea[Text Word] OR Neisseria gonorrhoeae[Mesh] OR gonococcus[Text Word] AND Drug Resistance, Bacterial[Mesh] OR antibacterial drug resistance[Text Word] AND Turkmenistan<br>Gonorrhea[Mesh] OR Gonorrhea[Text Word] OR Neisseria gonorrhoeae[Mesh] OR gonococcus[Text Word] AND Drug Resistance, Bacterial[Mesh] OR antibacterial drug resistance[Text Word] AND Russian Federation<br>Gonorrhea[Mesh] OR Gonorrhea[Text Word] OR Neisseria gonorrhoeae[Mesh] OR gonococcus[Text Word] AND Drug Resistance, Bacterial[Mesh] OR antibacterial drug resistance[Text Word] AND Ukraine<br>Gonorrhea[Mesh] OR Gonorrhea[Text Word] OR Neisseria gonorrhoeae[Mesh] OR gonococcus[Text Word] AND Drug Resistance, Bacterial[Mesh] OR antibacterial drug resistance[Text Word] AND Uzbekistan

SCOPUS<br><br>ALL(Gonorrhea) OR ALL("Neisseria gonorrhoeae") OR ALL(gonococcus) AND ALL(antibiotic drug resistance) OR ALL (antibacterial drug resistance) AND TITLE-ABS-KEY (Albania)<br>ALL(Gonorrhea) OR ALL("Neisseria gonorrhoeae") OR ALL(gonococcus) AND ALL(antibiotic drug resistance) OR ALL (antibacterial drug resistance) AND TITLE-ABS-KEY (Armenia)<br>ALL(gonorrhea) OR ALL("neisseria gonorrhoeae") OR ALL(gonococcus) AND ALL(antibiotic drug resistance) OR ALL (antibacterial drug resistance) AND TITLE-ABS-KEY(Azerbaijan)<br>ALL(gonorrhea) OR ALL("neisseria gonorrhoeae") OR ALL(gonococcus) AND ALL(antibiotic drug resistance) OR ALL (antibacterial drug resistance) AND TITLE-ABS-KEY(Belarus)<br>ALL(gonorrhea) OR ALL("neisseria gonorrhoeae") OR ALL(gonococcus) AND ALL(antibiotic drug resistance) OR ALL (antibacterial drug resistance) AND TITLE-ABS-KEY(Bosnia and Herzegovina)<br>ALL(gonorrhea) OR ALL("neisseria gonorrhoeae") OR ALL(gonococcus) AND ALL(antibiotic drug resistance) OR ALL (antibacterial drug resistance) AND TITLE-ABS-KEY(Georgia)<br>ALL(gonorrhea) OR ALL("neisseria gonorrhoeae") OR ALL(gonococcus) AND ALL(antibiotic drug resistance) OR ALL (antibacterial drug resistance) AND TITLE-ABS-KEY(Kazakhstan)<br>ALL(gonorrhea) OR ALL("neisseria gonorrhoeae") OR ALL(gonococcus) AND ALL(antibiotic drug resistance) OR ALL (antibacterial drug resistance) AND TITLE-ABS-KEY(Kosovo)<br>ALL(gonorrhea) OR ALL("neisseria gonorrhoeae") OR ALL(gonococcus) AND ALL(antibiotic drug resistance) OR ALL (antibacterial drug resistance) AND TITLE-ABS-KEY(Macedonia)<br>ALL(gonorrhea) OR ALL("neisseria gonorrhoeae") OR ALL(gonococcus) AND ALL(antibiotic drug resistance) OR ALL (antibacterial drug resistance) AND TITLE-ABS-KEY(Kyrgyzstan)<br>ALL(gonorrhea) OR ALL("neisseria gonorrhoeae") OR ALL(gonococcus) AND ALL(antibiotic drug resistance) OR ALL (antibacterial drug resistance) AND TITLE-ABS-KEY(Moldova)<br>ALL(gonorrhea) OR ALL("neisseria gonorrhoeae") OR ALL(gonococcus) AND ALL(antibiotic drug resistance) OR ALL (antibacterial drug resistance) AND TITLE-ABS-KEY(Montenegro)<br>ALL(gonorrhea) OR ALL("neisseria gonorrhoeae") OR ALL(gonococcus) AND ALL(antibiotic drug resistance) OR ALL (antibacterial drug resistance) AND TITLE-ABS-KEY(Serbia)<br>ALL(gonorrhea) OR ALL("neisseria gonorrhoeae") OR ALL(gonococcus) AND ALL(antibiotic drug resistance) OR ALL (antibacterial drug resistance) AND TITLE-ABS-KEY(Tajikistan)<br>ALL(gonorrhea) OR ALL("neisseria gonorrhoeae") OR ALL(gonococcus) AND ALL(antibiotic drug resistance) OR ALL (antibacterial drug resistance) AND TITLE-ABS-KEY(Turkey)<br>ALL(gonorrhea) OR ALL("neisseria gonorrhoeae") OR ALL(gonococcus) AND ALL(antibiotic drug resistance) OR ALL (antibacterial drug resistance) AND TITLE-ABS-KEY(Turkmenistan)<br>ALL(gonorrhea) OR ALL("neisseria gonorrhoeae") OR ALL(gonococcus) AND ALL(antibiotic drug resistance) OR ALL (antibacterial drug resistance) AND TITLE-ABS-KEY("Russian Federation")<br>ALL(gonorrhea) OR ALL("neisseria gonorrhoeae") OR ALL(gonococcus) AND ALL(antibiotic drug resistance) OR ALL (antibacterial drug resistance) AND TITLE-ABS-KEY(Ukraine)<br>ALL(gonorrhea) OR ALL("neisseria gonorrhoeae") OR ALL(gonococcus) AND ALL(antibiotic drug resistance) OR ALL (antibacterial drug resistance) AND TITLE-ABS-KEY(Uzbekistan)
WoSCC<br>TS=(Gonorrhea OR "Neisseria gonorrhoeae" OR gonococcus AND antibacterial drug resistance) AND CU=(Albania)<br>TS=(Gonorrhea OR "Neisseria gonorrhoeae" OR gonococcus AND antibacterial drug resistance) AND CU=(Armenia)<br>TS=(Gonorrhea OR "Neisseria gonorrhoeae" OR gonococcus AND antibacterial drug resistance) AND CU=(Azerbaijan)<br>TS=(Gonorrhea OR "Neisseria gonorrhoeae" OR gonococcus AND antibacterial drug resistance) AND CU=(Belarus)<br>TS=(Gonorrhea OR "Neisseria gonorrhoeae" OR gonococcus AND antibacterial drug resistance) AND CU=(Bosnia and Herzegovina) – 0<br>TS=(Gonorrhea OR "Neisseria gonorrhoeae" OR gonococcus AND antibacterial drug resistance) AND CU=(Georgia)<br>TS=(Gonorrhea OR "Neisseria gonorrhoeae" OR gonococcus AND antibacterial drug resistance) AND CU=(Kazakhstan)<br>TS=(Gonorrhea OR "Neisseria gonorrhoeae" OR gonococcus AND antibacterial drug resistance) AND CU=(Kosovo)<br>TS=(Gonorrhea OR "Neisseria gonorrhoeae" OR gonococcus AND antibacterial drug resistance) AND CU=(Macedonia)<br>TS=(Gonorrhea OR "Neisseria gonorrhoeae" OR gonococcus AND antibacterial drug resistance) AND CU=(Kyrgyzstan)<br>TS=(Gonorrhea OR "Neisseria gonorrhoeae" OR gonococcus AND antibacterial drug resistance) AND CU=(Moldova)<br>TS=(Gonorrhea OR "Neisseria gonorrhoeae" OR gonococcus AND antibacterial drug resistance) AND CU=(Montenegro)<br>TS=(Gonorrhea OR "Neisseria gonorrhoeae" OR gonococcus AND antibacterial drug resistance) AND CU=(Serbia)<br>TS=(Gonorrhea OR "Neisseria gonorrhoeae" OR gonococcus AND antibacterial drug resistance) AND CU=(Tajikistan)<br>TS=(Gonorrhea OR "Neisseria gonorrhoeae" OR gonococcus AND antibacterial drug resistance) AND CU=(Turkey)

TS=(Gonorrhea OR "Neisseria gonorrhoeae" OR gonococcus AND antibacterial drug resistance) AND CU=(Turkmenistan)<br>TS=(Gonorrhea OR "Neisseria gonorrhoeae" OR gonococcus AND antibacterial drug resistance) AND CU=(Russia)<br>TS=(Gonorrhea OR "Neisseria gonorrhoeae" OR gonococcus AND antibacterial drug resistance) AND CU=(Ukraine)<br>TS=(Gonorrhea OR "Neisseria gonorrhoeae" OR gonococcus AND antibacterial drug resistance) AND CU=(Uzbekistan)

Supplementary Figure 1. Flow Chart

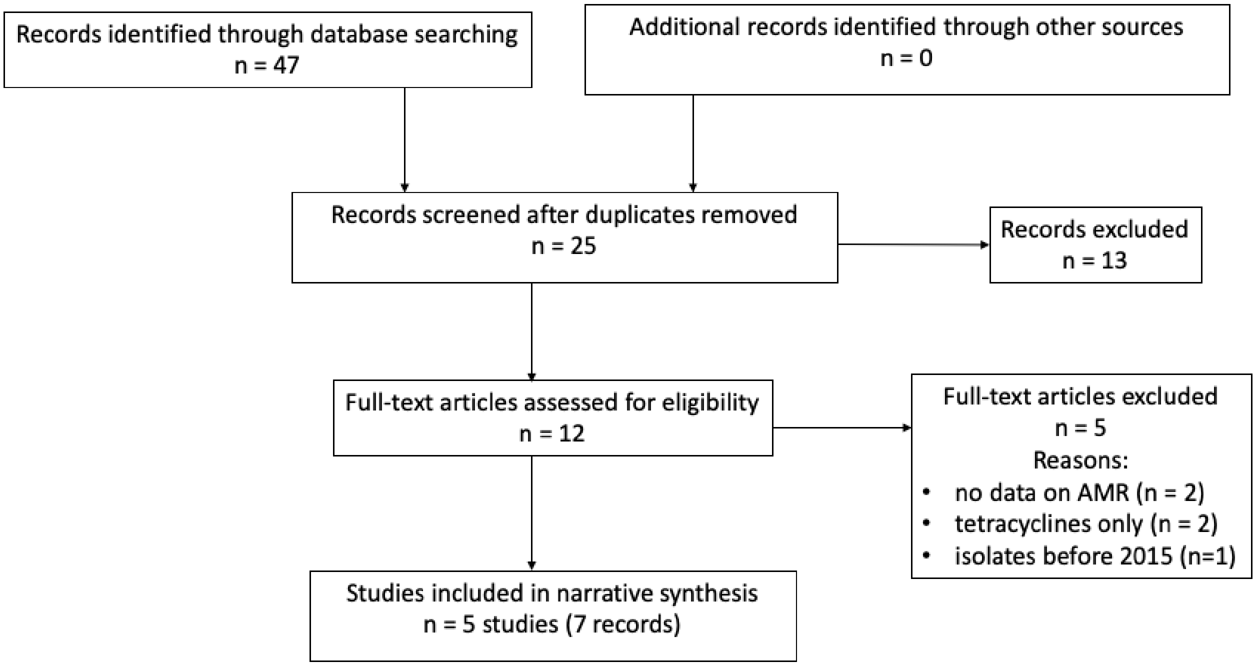

Supplementary Table 2. Data from the WHO Gonococcal Antimicrobial Surveillance Programme (GASP) and from published literature on number of gonococcal isolates tested for resistance to ceftriaxone, cefixime, extended-spectrum cephalosporines, azithromycin and ciprofloxacin from the non-EU/EEA countries of the WHO European Region, 2015-2018

	**GASP Data**					**Studies identified through systematic review**		
**Countries**	**Number of isolates per year**				**Comments**			
	**2015**	**2016**	**2017**	**2018**		**Study**	**Key study characteristics**	**Key study findings**
Belarus	-	-	36	19	DS/R to cefixime and extended-spectrum cephalosporins was 15.8% in 2018 and 22.2% in 2017, respectively. Proportion of isolates with resistance to ciprofloxacin was 30.6% in 2017 and 36.8% in 2018	No relevant studies identified	-	-
Georgia	-	-	-	-	-	Washington 2018	Case report, 2017, urine and urethral specimen from a 23 year old heterosexual M on military duty, client of a sex worker	R to cefixime and ciprofloxacin/quinolones
Kyrgyzstan	-	72	-	-	No isolates with DS/R to cefixime and to extended-spectrum cephalosporins or ciprofloxacin	No relevant studies identified	-	-
Russian Federation	293[a]	293[b]	-	-	No isolates with DS/R to cefixime, extended-spectrum cephalosporins and ciprofloxacin	Kubanov 2016	Design not specified, 2015, M and F, urethral and cervical specimens, n = 124	1/124 (0.8%) R to ceftriaxone, 2/124 (1.6%) R and 4 (3.2%) IS to azithromycin, 50/124 (40.3%) R and 1/124 (0.8%) IS to ciprofloxacin
						Kubanov 2019	Trends analysis (2005-2016), M and F, urethral and cervical specimens, n = 268 in 2016	2016: 0 (0%) R to ceftriaxone, 245/268 (91.4%) susceptible to azithromycin, R+IR not reported explicitly (implicitly 8.6%); 83/268 (31%) R, 2/268 (0.74%) IS to ciprofloxacin
						Shaskolskiy 2019	Design not specified, 2015-2017, M and F, urethral and cervical specimens, n = 522	1/522 R according to EUCAST criteria, 2/522 IS to ceftriaxone
Ukraine	33	25	14	10	10% of isolates were resistant to ciprofloxacin in 2018, and 21.4% in 2017. No isolates with DS/R to cefixime and to extended-spectrum cephalosporins	Boiko 2019	M and F, age not specified, urethral and cervical specimens, 2015: n=33, 2016: n=25, 2017: n=13, 2018: n =10 (corresponding to GASP apart from 2017)	Resistance reported for the period 2013-2018 in a total of 150 specimens.

DS = decreased susceptibility; IS = intermediate susceptibility; R= resistance. M= male, F = female, n = number of specimens. None of the four countries reported isolates with R to azithromycin, nor DS/R to ceftriaxone to GASP during the observation period. [a]tested for azithromycin and ciprofloxacin only [b]tested for cefixime and extended spectrum-cephalosporines only

Supplementary Table 3. Reasons for exclusion of identified studies for which full text was assessed

Study ID	Reason(s) for exclusion and comments
Brinda 2020	No data on AMR in *Neisseria gonorrhoeae.*
Dementieva 2019	Only data on AMR to tetracyclines in *Neisseria gonorrhoeae*. Possibly same isolates as Shaskolsky 2018 analysed and reported.
Shaskolsky 2018	Only data on AMR to tetracyclines in *Neisseria gonorrhoeae*. Possibly same isolates as Dementieva 2019 analysed and reported
Lebedzeu 2015	Isolates before 2015 included
Shaskolskiy 2020	No data on DS/R included, previous reports Kubanov 2019, Shaskolsky 2019

AMR = antimicrobial resistance; DS = decreased susceptibility; R= resistance

Supplementary Table 4. Quality assessment of included studies

Criteria	Number of studies meeting criteria out of all studies included (percentage)
1. Is the research design described?	2/5 (40%)
2. Does the study state the period of time during which samples were specifically collected?	4/5 (80%)
3. Is the setting of the study and data acquisition clearly described?	4/5 (80%)
4. Is the study population clearly described (males, females, age groups, risk groups, comorbidities?)	0/5 (0%)
5. Are the criteria for enrolment in the study clearly stated?	0/5 (0%)
6. Is there a clear description of the types of specimen collected and how these were collected, in particular in regard to body site (urethral, cervical, urine, rectal, pharyngeal)?	5/5 (100%)
7. Did the study describe the overall number of samples collected by the following categories:<br>Sex<br>Age<br>Risk group<br>Anatomical site that specimens are taken from (urethral, cervical, urine, rectal, pharyngeal)	1/5 (20%)<br>5/5 (100%)<br>1/5 (20%)<br>1/5 (20%)<br>1/5 (20%)
8. Does the study describe the number of samples tested and indicate reasons for exclusion, if any?	2/5 (40%)
9. Is there description of how specimens were handled, transported and stored after collection?	4/5 (80%)
10. Are the media for culture described?	4/5 (80%)
11. Did the study describe the total number of isolates?	5/5 (100%)
12. Does the study describe isolates by source (anatomical site, i.e. urethral, cervical, urine, rectal, pharyngeal)?	0/5 (0%)
13. Does the study describe isolates by category?<br>Sex<br>Age<br>Risk group<br>Body site (urethral, cervical, urine, rectal, pharyngeal)	1/5 (20%)<br>4/5 (80%)<br>1/5 (20%)<br>1/5 (20%)<br>1/5 (20%)
14. Does the study describe the type of susceptibility testing used?	5/5 (100%)
15. Did the study use any internal quality control measures (example, reference strains)?	5/5 (100%)
16. Did the study specify the testing standard used<br>-EUCAST<br>-Other	5/5 (100%)<br>3/5 (60%)<br>2/5 (40%)
17. Did the study indicate if quality testing procedures were used (e.g. confirmatory testing by independent lab) and concordance level?	0/5 (0%)
18. Did the study calculate the frequency of resistance to an antibiotic as the total number of resistant isolates divided by the total number of isolates tested with a given antibiotic?	4/5 (80%)

Supplementary Table 5. Contextual factors by countries

Country	Population (in thousands, in 2020)	EU membership candidate country[a]	Healthcare expenditure per capita in 2018 in current $US[b]	Out of pocket expenditure in 2018 (%)[c]	Number of public STI laboratories[d]	Regional reference STI laboratories[d]	National STI reference laboratories[d]	Expert laboratories[d]
Belarus	9453	No	365.25	24.95	8	No	Syph	Gon
Georgia	3989	No	317.75	47.67	7	No	No	No
Kazakhstan	18777	No	275.85	33.47	Not known	No	No	No
Montenegro	628	Yes	731.48	39.61	1	No	Syph; Gon, Chla	No
North Macedonia	2083	Yes	399.10	42.11	No response	No response	No response	No
Serbia	8737	Yes	617.09	38.31	Not known	No	Syph	No
Ukraine	43734	No	228.39	49.35	55	Syph; Gon, Chla	Syph	No

Syph = syphilis; Gon= gonorrhoea, Chla = chlamydia
[a] Available from: https://ec.europa.eu/neighbourhood-enlargement/countries/check-current-status_en (accessed on July18, 2021);
[b]Available from: https://data.worldbank.org/indicator/SH.XPD.CHEX.PC.CD (accessed on July18, 2021);
[c] % of current health expenditure; https://data.worldbank.org/indicator/SH.XPD.OOPC.CH.ZS?name_desc=false (accessed on July18, 2021);
[d] Information obtained through the questionnaire (Supplementary Material 1)
